# Efficacy of five peroral therapeutics against *Ichthyophthirius multifiliis* infestation in salmonids

**DOI:** 10.17221/99/2025-VETMED

**Published:** 2026-07-23

**Authors:** Katerina Matejickova, Jitka Motlova, Hana Novotna, Zuzana Mikulkova, Pavlina Ginterova, Martin Jerabek, Lubomir Pojezdal

**Affiliations:** ^1^Department of Infectious Diseases and Preventive Medicine, Veterinary Research Institute, Brno, Czech Republic; ^2^Tekro, spol. s r.o., Prague, Czech Republic

**Keywords:** amprolium, brook trout, clopidol, doxycycline, praziquantel, toltrazuril

## Abstract

Abstract: *Ichthyophthirius multifiliis* is a widespread parasitic ciliate causing significant losses in intensive freshwater aquaculture. This study evaluated the efficacy of medicated feed containing amprolium hydrochloride (2 mg/kg b.w./day), clopidol (2 mg/kg b.w./day), praziquantel (20 mg/kg b.w./day), toltrazuril (50 mg/kg b.w./day), and doxycycline (40 mg/kg b.w./day) administered at different stages of infection in experimentally infected brook trout fingerlings. Doxycycline and praziquantel demonstrated high protective efficacy (0–5% mortality) when applied during parasite exposure or the preclinical stage of infection. In contrast, amprolium hydrochloride, toltrazuril, and clopidol showed limited or no protective effect. The timing of administration was critical, and prophylactic use of effective compounds via feed may offer a practical strategy for controlling *I. multifiliis* outbreaks. These findings support the potential of doxycycline and praziquantel as oral chemotherapeutics in aquaculture, warranting further investigation for therapeutic use in advanced infection stages.

Ichthyophthiriasis caused by the protozoan *Ichthyophthirius multifiliis* (Ich) is one of the major parasitic diseases of freshwater fish worldwide. It is associated with high mortality in juvenile fish due to its ability to invade the epidermis and gill epithelium, leading to substantial economic losses, especially in intensive aquaculture systems of freshwater salmonids (Buchmann et al. 2004). For decades, malachite green and dimetridazole were used effectively against Ich. However, their use in food-producing animals has been banned due to their carcinogenic, teratogenic and mutagenic properties (European Commission 2023a).

Currently, control of Ich relies mainly on chemical treatments. These predominantly affect the free-swimming stages of the parasite (theronts and tomonts) and therefore require multiple applications to achieve effective control (Rach et al. 2000). Additionally, these compounds have low therapeutic indices and may potentially damage the gills and epidermis of fish, increasing susceptibility to secondary infections caused by bacteria (e.g., *Aeromonas* spp., *Flavobacterium* spp.) and other parasites (Buchmann et al. 2004). Protocols relying on water disinfection based on hydrogen peroxide, sodium percarbonate, and peracetic acid have been tested, but their efficacy remains inferior to that of malachite green (Buchmann et al. 2003; Chupani et al. 2014).

Compared with immersion treatment or water disinfection, oral administration of chemotherapeutics offers several potential advantages, including reduced stress, easier handling, prolonged activity within fish tissues, and independence from environmental water parameters, such as pH, conductivity, and organic load (Shinn et al. 2003).

In a study by Shinn et al. (2003), amprolium hydrochloride and clopidol, drugs routinely used to control coccidiosis in poultry, both provided significant protection against subsequent exposure to Ich theronts in rainbow trout fingerlings. However, their efficacy in reducing Ich trophont numbers was lower when administered after infection. Toltrazuril, another anticoccidial compound used in farm animals, was reported by Jaafar and Buchmann (2011) to reduce Ich infection in juvenile rainbow trout when administered at a concentration of 2.5 mg/g feed for three consecutive days prior to exposure to theronts, but it was ineffective when administered post-infection. None of these compounds is specifically licensed for use in fish in the EU, but they may be used according to the cascade system (European Parliament and Council of the European Union 2019). Praziquantel is a broad-spectrum anthelmintic widely used in both human and veterinary medicine and has been proven effective against a range of platyhelminth parasites in fish (Bader et al. 2019). Its use in the EU is approved with maximum residue limits (MRLs) for finfish in muscle and skin at 20 µg/kg (European Commission 2023b). Doxycycline is a broad-spectrum tetracycline antibiotic used in the treatment of infections caused by Gram-positive and Gram-negative bacteria, mycoplasmas, chlamydiae, rickettsiae and protozoan parasites in humans and livestock. In aquaculture, doxycycline is recommended to be used at a dose of 20 mg/kg body weight (b.w.) for 3–5 days to treat bacterial infections (Corum et al. 2023).

The aim of this study is to evaluate the potential efficacy of medicated feed containing five antiparasitic and antimicrobial substances, commonly approved for use in farm animals in the EU and the Czech Republic. Specifically, the study investigates their effectiveness at different stages of Ich infection in brook trout, while also considering practical applicability and potential regulatory restrictions in aquaculture systems.

## MATERIAL AND METHODS

### Source of infection

All experiments were approved by the Central Commission for Animal Welfare of the Ministry of Agriculture of the Czech Republic (Permission No. MZe 2407). Juvenile rainbow trout (*Onco-rhynchus mykiss*), naturally infected with *I. multifiliis* but free of other ectoparasites, were obtained from a commercial fish farm and used as the source of infection. The Ich population was maintained in the laboratory by serial passage to naïve rainbow trout.

### Experimental fish

Brook trout fingerlings (*Salvelinus fontinalis*) were obtained from a commercial hatchery. The absence of ectoparasites was confirmed in five individuals by light microscopy of skin mucus and gill samples, and the same samples were further confirmed as Ich-negative by real-time PCR (Jousson et al. 2005). Fish were acclimated for at least 14 days prior to experimentation.

### Preparation and administration of medicated feed

Medicated feeds were prepared manually by supplementing a commercial pelleted diet (INICIO Plus M, 0.8 mm; BioMar Group, Denmark) with the following chemotherapeutics: amprolium hydrochloride (AMP, 0.1 mg/g feed), clopidol (CLP, 0.1 mg/g feed), praziquantel (PZQ, 1 mg/g feed), toltrazuril (TLZ, 2.5 mg/g feed), or doxycycline (DOX, 2 mg/g feed). Except for PZQ, the substances were dissolved in distilled water and sprayed onto the pellets. Due to insolubility in water, PZQ was incorporated into the pellets with the addition of rapeseed oil. Control fish were fed an identical diet without additives. All groups were fed once daily at 2% of body weight for 10 consecutive days, and feed intake was confirmed visually.

### Experimental infection design

Fish were exposed to Ich by 24-hour cohabitation with infected rainbow trout. Afterwards, fish were randomly distributed into six groups (five treatments and one untreated control) in 50 l aquaria with a closed water recirculation system under a 12 h light/12 h dark cycle, supplied with carbon-filtered, dechlorinated tap water maintained at 12 ± 1 °C, pH 7.0 ± 0.4, and dissolved oxygen >5.5 mg/l.

Three separate sequential experimental trials were conducted to evaluate a medicated feed administered at different stages of Ich infection. In Experiment 1, a ten-day treatment period was initiated at the time of exposure to Ich. The mean fish weight was 2.5 g, *n* = 20 fish per group, with six separate groups: Control, AMP, CLP, DOX, PZQ, TOL (120 fish in total). In Experiment 2, the ten-day treatment was initiated seven days post-infection, the mean fish weight was 6.0 g, with *n* = 8 fish per group and the same six groups as in Experiment 1 (48 fish in total). In Experiment 3, the ten-day treatment was initiated at the onset of mortality, following a microscopic examination of five fish that confirmed the presence of Ich trophonts (1–10 trophonts per mucus smear from one lateral side of each fish), with a mean fish weight of 9.0 g and *n* = 20 fish per group, with only three groups: Control, DOX, and PZQ (60 fish in total). Each experiment lasted for 32 days.

### Monitoring and sampling

Fish mortalities, water temperature, oxygen saturation, and pH were recorded daily. Mucus samples from dead fish were collected by scraping and examined microscopically to confirm the presence of Ich trophonts (data not provided). At the end of the experimental trials, all surviving fish were euthanised by an overdose of benzocaine. Additionally, in Experiment 3, two fish per group were randomly selected, and examined microscopically for Ich trophont counts in mucus at three time points – prior to treatment, one day post-treatment, and 15 days post-treatment. Two fish and water samples from Experiment 3 were also subsequently stored at –80 °C for pharmacological analyses.

### Liquid chromatography-mass spectrometry

The concentrations of DOX and PZQ in whole fish homogenates and water samples from Experiment 3 were determined using an Agilent 1290 Infinity liquid chromatograph coupled with an Agilent 6460 TripleQuad LC/MS mass spectrometer (Agilent, Santa Clara, CA, USA). Samples were separated by a Luna Omega C18 column (1.6 µm; 2.1 × 100 mm) at 40 °C. The mobile phase consisted of 0.1% formic acid in water and 0.1% formic acid in methanol at a flow rate of 0.35 ml/min. Sample preparation was based on a previously published QuEChERS extraction method (Kim et al. 2021), with slight modifications [see Electronic Supplementary Material (ESM)]. Mean concentrations of DOX and PZQ in tissue and water samples were calculated from the detected values. The data were evaluated to assess the persistence of DOX and PZQ in fish tissues and the aquatic environment at one and 15 days after the ten-day administration of medicated feeding.

### Statistical analysis

Survival analysis was conducted using the Kaplan–Meier method to estimate mortality functions. Differences in survival between experimental groups were evaluated using the Mantel–Haenszel log-rank test. All statistical analyses were performed in R (v4.5.1; R Project for Statistical Computing, Austria) using the *survival* and *survminer* packages. Kaplan–Meier mortality curves were visualised with the *ggplot2* package. A significance level of α = 0.05 was considered for all statistical tests. Mortality prediction was assessed using a *Z*-statistic, calculated as:

Z=(O−E)/√var(O−E) (1)

where:

*O* – the observed number of mortalities;

*E* – the expected number;

var(*O* − *E*) – the variance of the difference between observed and expected values.

## RESULTS

**Figure 1:**
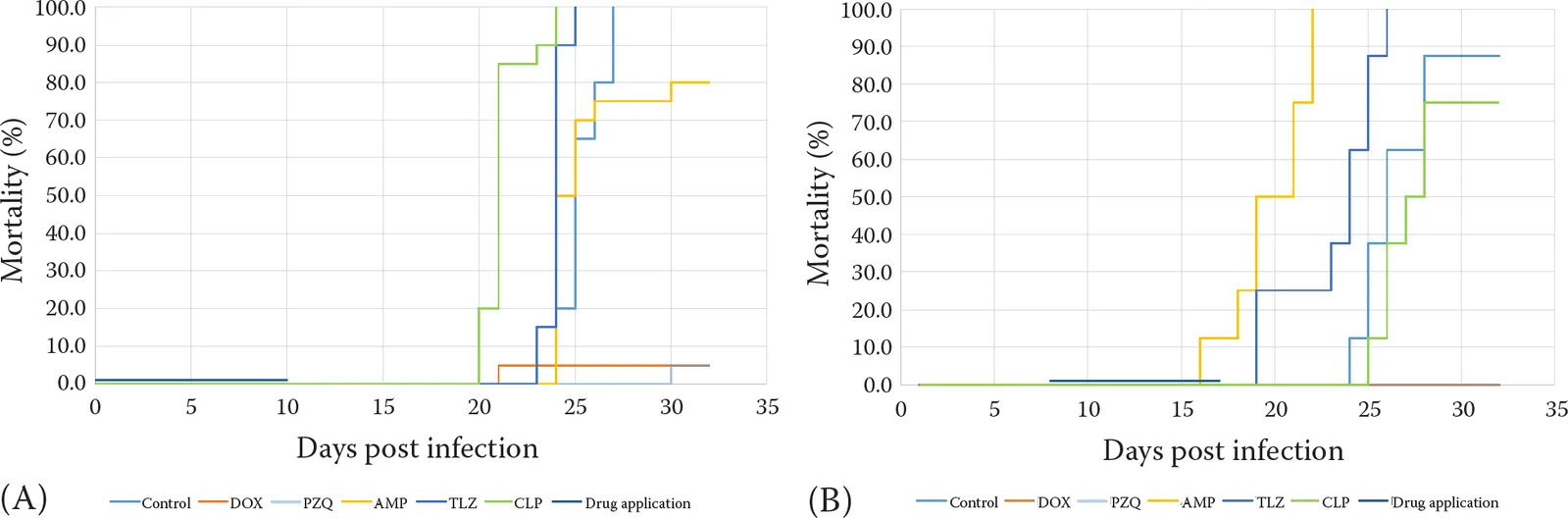
Kaplan–Meier mortality curves for brook trout infected with *Ichthyophthirius multifiliis* and treated with a medicated diet for 10 days (A) Experiment 1: Treatment initiated at the time of exposure to Ich; (B) Experiment 2: Treatment initiated 7 days post-infection AMP = amprolium; CLP = clopidol; DOX = doxycycline; PZQ = praziquantel; TLZ = toltrazuril

In Experiment 1 (Table 1, Figure 1A), where treatment was initiated at the time of exposure to Ich, the lowest mortality was observed in fish fed DOX and PZQ diets (5% each). Both treatments differed significantly from the Control group (*P* < 0.000 1), showing negative *Z*-stat values of mortality prediction, indicating a strong survival benefit. No statistically significant difference was found between the DOX and PZQ groups.

**Table 1:** Effect of medicated feed on the mortality of brook trout infested with *Ichthyophthirius multifiliis*

Diet type	Compound (dose)	Mortality (%)	Significance, *P*-value, omnibus test (χ^2^) stat	Mortality prediction *Z*-stat
**Experiment 1**: Treatment was initiated at the time of exposure to Ich (early infection stage in brook trout; mean body weight 2.5 g; water temperature 12 ± 1 °C; *n* = 20 fish) and administered for 10 days				
DOX	doxycycline (40 mg/kg b.w./day)	5	Sig. (*P* < 0.000 1)	–5.15
PZQ	praziquantel (20 mg/kg b.w./day)	5	Sig. (*P* < 0.000 1)	–5.32
AMP	amprolium hydrochloride (2 mg/kg b.w./day)	80	ns	0.83
TLZ	toltrazuril (50 mg/kg b.w./day)	100	Sig. (*P* < 0.000 1)	4.10
CLP	clopidol (2 mg/kg b.w./day)	100	Sig. (*P* < 0.000 1)	10.41
Control		100		1.76

**Experiment 2**: Treatment was initiated during the preclinical stage (7 days post-infection; mean body weight 6.0 g; water temperature 12 ± 1 °C; *n* = 8 fish) and administered for 10 days				
DOX	doxycycline (40 mg/kg b.w./day)	0	Sig. (*P* = 0.001)	–3.19
PZQ	praziquantel (20 mg/kg b.w./day)	0	Sig. (*P* = 0.001)	–3.19
AMP	amprolium hydrochloride (2 mg/kg b.w./day)	100	Sig. (*P* = 0.000 2)	7.05
TLZ	toltrazuril (50 mg/kg b.w./day)	100	Sig. (*P* = 0.012 2)	3.46
CLP	clopidol (2 mg/kg b.w./day)	75	ns	0.02
Control		88		0.90

**Experiment 3**: Treatment was initiated at the onset of mortality (mean body weight 9.0 g, water temperature 12 ± 1 °C, *n* = 19–20 fish) and administered for 10 days				
DOX	doxycycline (40 mg/kg b.w./day)	20	ns	0.29
PZQ	praziquantel (20 mg/kg b.w./day)	20	ns	0.13
Control		16		–0.42

Statistical significance was assessed by Kaplan–Meier survival analysis compared with the Control group. Mortality prediction: Negative *Z*-stat values indicate a survival advantage relative to the Control group, while positive values correspond to higher mortality risk

ns = not significant (*P* > 0.05); Sig. = significant (level of significance)

In contrast, TLZ and CLP diets resulted in complete mortality (100%) and exhibited highly significant but positive *Z*-stat values, suggesting a detrimental effect on fish survival. The AMP group exhibited 80% mortality, which did not differ significantly from the Control group. The Control group reached 100% mortality.

In Experiment 2 (Table 1, Figure 1B), where treatment was applied during the preclinical stage (7 days post-infection), complete survival (0% mortality) was achieved in both DOX and PZQ groups. Kaplan–Meier survival analysis showed the curves of both diets differing significantly from the Control and all other diets (*P* < 0.001), with negative *Z*-stat values indicating improved survival. No statistically significant difference was found between the DOX and PZQ groups. The AMP and TLZ groups exhibited 100% mortality and the CLP group 75% mortality with no significant difference from the Control group. The Control group experienced 88% mortality, confirming that the infection progressed in the absence of effective treatment.

In Experiment 3 (Table 1), only DOX and PZQ diets were tested. Treatment was initiated at the onset of mortality, following a microscopic examination of five fish that confirmed the presence of Ich trophonts (1–10 trophonts per mucus smear from one lateral side of each fish). Overall mortality in this experiment remained low across all groups, including the Control, and no statistically significant differences were observed between treatments (*P* > 0.05). However, quantitative assessment of parasite load revealed pronounced differences in infection intensity. One day post-treatment, the number of Ich trophonts per lateral body side was 55 in the Control group, 0 in the DOX group, and 8 in the PZQ group. Fifteen days post-treatment, parasite numbers markedly declined in all groups (Control 2, DOX 0, PZQ 2), indicating near-complete recovery (Table 2).

**Table 2:** Effect of medicated feeds on *Ichthyophthirius multifiliis* infection in brook trout with treatment initiated at the onset of mortality (Experiment 3)

Days post-treatment	Diet type (dose)	Concentration in fish (µg/kg)	Concentration in water (µg/l)	No. of trophonts on one body side of fish*
1	doxycycline (40 mg/kg b.w./day)	>2 000	41.7	0
		>2 000		
15		45.6	13.8	0
		<50.0		
				
1	praziquantel (20 mg/kg b.w./day)	162.1	88.6	8
		633.5		
15		<50.0	48.8	2
		<50.0		
				
1	Control	na	na	55
15		na		2

The concentrations of doxycycline and praziquantel in whole fish homogenates and aquarium water after 10 days of oral administration. Parasite load is expressed as the number of Ich trophonts on one lateral body side of each fish

*Two fish per group were sampled

na = not applicable

Liquid chromatography-mass spectrometry analyses confirmed that both DOX and PZQ reached quantifiable concentrations in fish tissues after the end of treatment. DOX concentrations in whole fish homogenates exceeded 2 000 µg/kg on Day 1 post-treatment, while PZQ reached a mean concentration of 398 µg/kg. Concentrations of both compounds declined to below 50 µg/kg by Day 15 after the last administration. Corresponding water concentrations were markedly lower, decreasing from 41.7 µg/l on Day 1 to 13.8 µg/l on Day 15 for DOX and from 88.6 µg/l to 48.8 µg/l for PZQ (Table 2). These results demonstrate efficient oral uptake and systemic distribution of both compounds, accompanied by a rapid decline in environmental residues over time.

## DISCUSSION

This study aimed to evaluate the potential efficacy of medicated feeds for the control of ichthyophthiriasis in experimentally infected juvenile brook trout (*Salvelinus fontinalis*), treated with five chemotherapeutics at three distinct stages of infection.

The results demonstrated that treatment efficacy was highly dependent on both the type of chemotherapeutic agent and the timing of administration. Amprolium hydrochloride, toltrazuril, and clopidol exhibited minimal or no protective effects in any of the experimental regimes, despite previous reports of efficacy in rainbow trout (Shinn et al. 2003; Jaafar and Buchmann 2011; Abdel-Hafez et al. 2014). In contrast, doxycycline and praziquantel were highly effective when administered either at the time of parasite exposure or during the preclinical stage of infection, resulting in almost complete protection. These findings suggest that both compounds may disrupt early developmental stages of the parasite or enhance host defence mechanisms before the onset of severe infection.

Treatment initiated after the onset of mortality did not significantly improve survival in this study, but additional parasitological and residue data provide important context for interpreting this outcome. Although only two fish per group were analysed, and further research with a statistically robust sample size is therefore needed, trophont counts one day post-treatment were markedly reduced in fish receiving DOX or PZQ, whereas the control fish exhibited heavy infestation (55 trophonts). This suggests effective suppression of Ich development by both DOX and PZQ, even though this therapeutic effect was not reflected in survival outcomes. By Day 15, infection intensity had decreased across all groups, reaching 0–2 trophonts per fish, indicating almost complete recovery. Quantitative residue analyses confirmed high initial concentrations of both active substances in fish tissues, demonstrating efficient oral uptake. Although drug concentrations in water were low and decreased rapidly over time, suggesting that the therapeutic effect was primarily associated with systemic absorption rather than environmental exposure, the effects of DOX and PZQ on the free-swimming and encysted stages of the parasite warrant further investigation. The maintenance of measurable tissue concentrations throughout the treatment supports the biological activity of both drugs even though mortality differences were not statistically significant. These findings collectively indicate that the apparent absence of treatment effect in Experiment 3 was not attributable to reduced drug efficacy, but rather to the mild course of infection at this stage, combined with increased fish resilience (larger body weight, improved condition) and transfer to clean aquaria prior to treatment, which likely facilitated the spontaneous resolution of infection even in the untreated group.

Abdel-Hafez et al. (2014) also demonstrated optimal reduction of Ich infestation in juvenile rainbow trout fed a diet containing DOX combined with toltrazuril. Although DOX exhibits antiprotozoal activity, its mechanism of action against protozoa remains unclear. It is hypothesised that DOX targets the parasite’s ribosomes, which share structural similarity with bacterial ribosomes, thereby inhibiting protein synthesis. Its antibacterial activity may also contribute to its efficacy against Ich, since endosymbiotic rickettsial bacteria have been observed within trophonts and theronts, and extracellular flavobacteria have been found on the surface of theronts and tomonts, where they interact with cilia and mucus and may exacerbate infection severity (Sun et al. 2009).

Doxycycline possesses a long elimination half-life, good oral absorption, high lipophilicity and tissue permeability, making it a strong candidate for oral administration. Since DOX is not specifically authorised for use in fish within the EU, its administration in aquaculture should be considered off-label and must be performed under strict veterinary supervision, in full compliance with the established MRL (100 µg/kg in muscle and skin in natural proportions), withdrawal periods and safety estimation for the target fish species (European Commission 2015). For rainbow trout treated with 20 mg/kg DOX for five days, the withdrawal period required to ensure safe consumption of edible tissues (muscle and skin) is at least 35 days at 10 °C and 31 days at 17 °C (Corum et al. 2023).

Praziquantel has been successfully used to control a broad spectrum of platyhelminthes in fish (Bader et al. 2019). Its mechanism of action involves binding to voltage-gated Ca^2+^ channels and disrupting calcium homeostasis. PZQ also damages the protective layer of the parasites (tegument), causing vacuolisation and cell degeneration. It may also affect ciliates due to their reliance on Ca^2+^-dependent signalling pathways that regulate ciliary motion and contractile vacuole activity, similar to its effect on helminths (Doenhoff et al. 2008). However, clarification of its precise mechanism of action in protozoans requires further investigation. An advantage of PZQ is that its safety profile and pharmacological properties are already well established, which allows its controlled and justified use in clearly indicated cases.

Both doxycycline and praziquantel can persist in the aquatic environment, even at low residual concentrations, and are reported as potential environmental contaminants affecting aquatic organisms and microbial communities. Their use should therefore not replace non-antibiotic treatments and must follow the principles of responsible drug management (FAO 2019).

In conclusion, the successful control of *I. multifiliis* using orally administered chemotherapeutics depends not only on the selection of effective compounds but also on the timing of their application. DOX and PZQ demonstrated strong prophylactic potential, whereas their therapeutic efficacy in advanced stages of infection warrants further targeted investigation.

## Supplementary Material

Supplementary Files
