# Supplementary material for: Efficacy of five peroral therapeutics against Ichthyophthirius multifiliis infestation in salmonids
Source: Vet Med (Praha). 2026 Jul 23;71(7):255–62. doi: 10.17221/99/2025-VETMED (PMC13501266; doi:10.17221/99/2025-VETMED)
Supplement: Supplementary file 1 [file VETMED-71-07-125099-s001.pdf]

https://doi.org/10.17221/99/2025-VETMED

Efficacy of five peroral therapeutics against *Ichthyophthirius multifiliis* infestation in salmonids

Katerina Matejickova[1], *Jitka Motlova*[1], *Hana Novotna*[1], *Zuzana Mikulkova*[2], *Pavlina Ginterova*[2], *Martin Jerabek*[2], *Lubomir Pojezdal*[1]

[1]*Department of Infectious Diseases and Preventive Medicine, Veterinary Research Institute, Brno, Czech Republic*

[2]*Tekro, spol. s r.o., Prague, Czech Republic*

**Corresponding author: lubomir.pojezdal@vri.cz*

The authors are fully responsible for both the content and the formal aspects of the electronic supplementary material. No editorial adjustments were made.

Electronic Supplementary Material (ESM)

Preparation of fish tissue samples for liquid chromatography-mass spectrometry

Briefly, after thawing at room temperature, a 0.5 g portion of the homogenised sample was mixed with 20 µl of methacycline working solution (internal standard for DOX and PZQ, approx. 10 ppm) and 2.1 ml of HPLC-grade water. The sample was vortexed for 5 min at 2 500 rpm. Subsequently, 2.5 ml of extraction solution (1% formic acid in acetonitrile) was added and vortexed again (1 min at 2 500 rpm). Then, 0.3 g of NaCl and 1.0 g of $MgSO_4$ were added, followed by vigorous vortexing (1 min at 2 500 rpm) and centrifugation in a sealed tube (4 min at 1 500 × *g*). After centrifugation, 200 µl of the upper acetonitrile layer was transferred to a microvial for analysis. Each sample was prepared once and analysed in duplicate.

Water samples were thawed at room temperature, filtered through a 0.45 µm syringe filter, transferred to a microvial, and analysed directly. Detection was performed at a wavelength of 350 nm.

The calibration standards of doxycycline hyclate (DOX, purity > 95%) and praziquantel (PZQ, purity > 95%) were prepared at five calibration levels, and each calibration point was prepared once and analysed twice. Tissue samples (whole fish homogenate) from untreated fish were spiked with a reference standard solution of DOX and PZQ at the same final concentration of 50, 200, 500, 1 000 and 2 000 µg kg^{-1} (limit of detection, LOQ < 50 µg kg^{-1}). Water samples from aquaria of untreated fish were spiked with a standard solution of DOX and PZQ to yield concentrations of 2, 10, 50, 100, 200 µg l^{-1} (LOQ < 2 µg l^{-1}). Samples were prepared and analysed as described above.
